# Supplementary material for: Identifying the Multitarget Pharmacological Mechanism of Action of Genistein on Lung Cancer by Integrating Network Pharmacology and Molecular Dynamic Simulation
Source: Molecules. 2024 Apr 23;29(9):1913. doi: 10.3390/molecules29091913 (PMC11085736; doi:10.3390/molecules29091913)
Supplement: Supplementary file 1 [file molecules-29-01913-s001.zip › molecules-2925355-supplementary.pdf]

Supplementary Data

Identifying the multi-target pharmacological mechanism of action of genistein on lung cancer by integrating network pharmacology and molecular dynamics simulation

Table S1: Molecular docking and binding free energy results of Genistein and selected compounds.

Target-complex	Docking Score (kcal/mol)	MM-GBSA score (kcal/mol)
AKT1-Genistein	-8.89	-47.77
AKT1-MK2206	-8.50	-71.06
AKT1-H-89	-6.45	-51.18
EGFR-Genistein	-7.80	-42.30
EGFR-Afatinib	-8.38	-59.86
EGFR-Compound20a	-12.11	-92.90
STAT3-SD-36	-13.25	-86.33
STAT3-genistein	-4.33	-33.70

Table S2: 2D interactions of genistein and selected compounds with AKT1.

Compound Name	Hydrogen bond (length in Å)	Hydrophobic bond (length in Å)	π-cation
Genistein	Gln^{79}(2.22)<br>Ser^{205}(2.01)	Gln^{79}(3.88)<br>Leu^{210}(3.77)<br>Leu^{210}(3.77)	-----------
MK-2206	Asn^{53}(2.20)<br>Tyr^{272}(1.89)	Trp^{80}(3.77)<br>Trp^{80}(3.85)<br>Leu^{210}(3.81)<br>Leu^{264}(3.80)<br>Val^{270}(3.96)<br>Val^{270}(3.70)<br>Tyr^{272}(3.66)<br>Tyr^{272}(3.58)<br>Asp^{274}(3.77)	-----------
H-89	Gln^{79}(1.77)	Asn^{53}(3.95)<br>Gln^{79}(3.56)<br>Gln^{79}(3.92)<br>Trp^{80}(3.89)<br>Trp^{80}(3.59)<br>Leu^{210}(3.72)<br>Val^{270}(3.46)<br>Val^{270}(3.85)	Trp^{80}(3.74)<br>Trp^{80}(3.98)

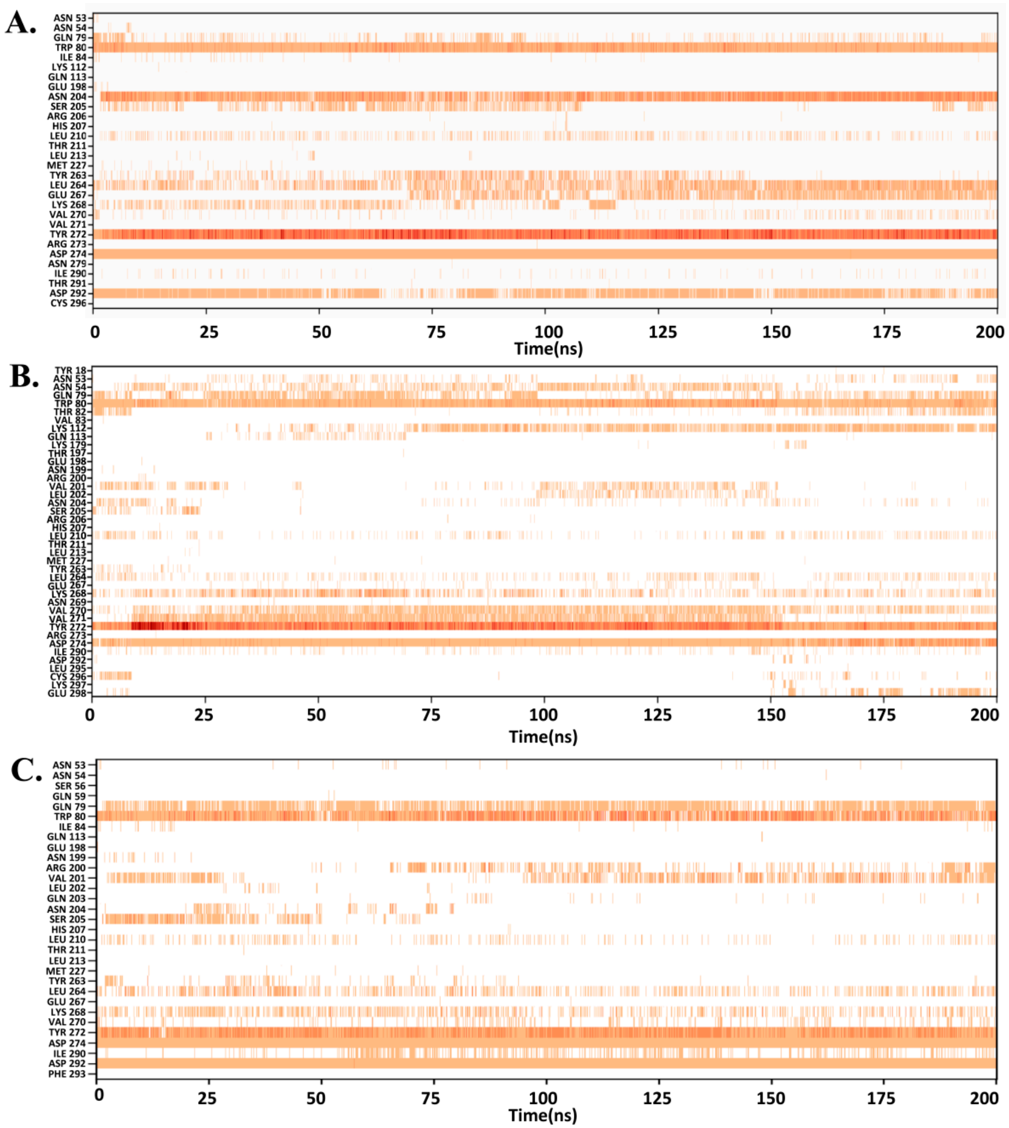

Figure S1: Time-dependent contacts between MK-2206 and AKT1 of **A)** Run 1, **B)** Run 2, and **C)** Run 3.

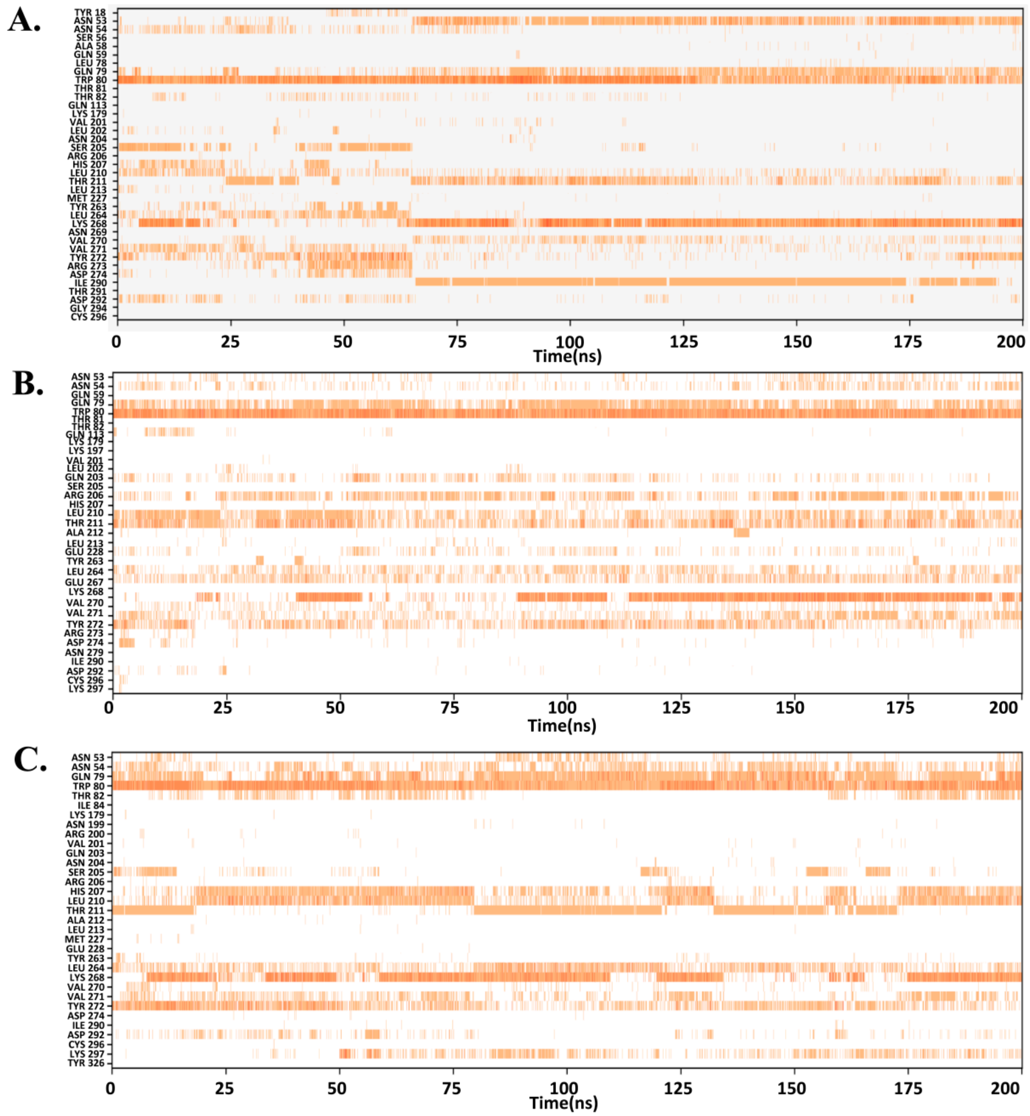

Figure S2: Time-dependent contacts between genistein and AKT1 of **A)** Run 1, **B)** Run 2, and **C)** Run 3.

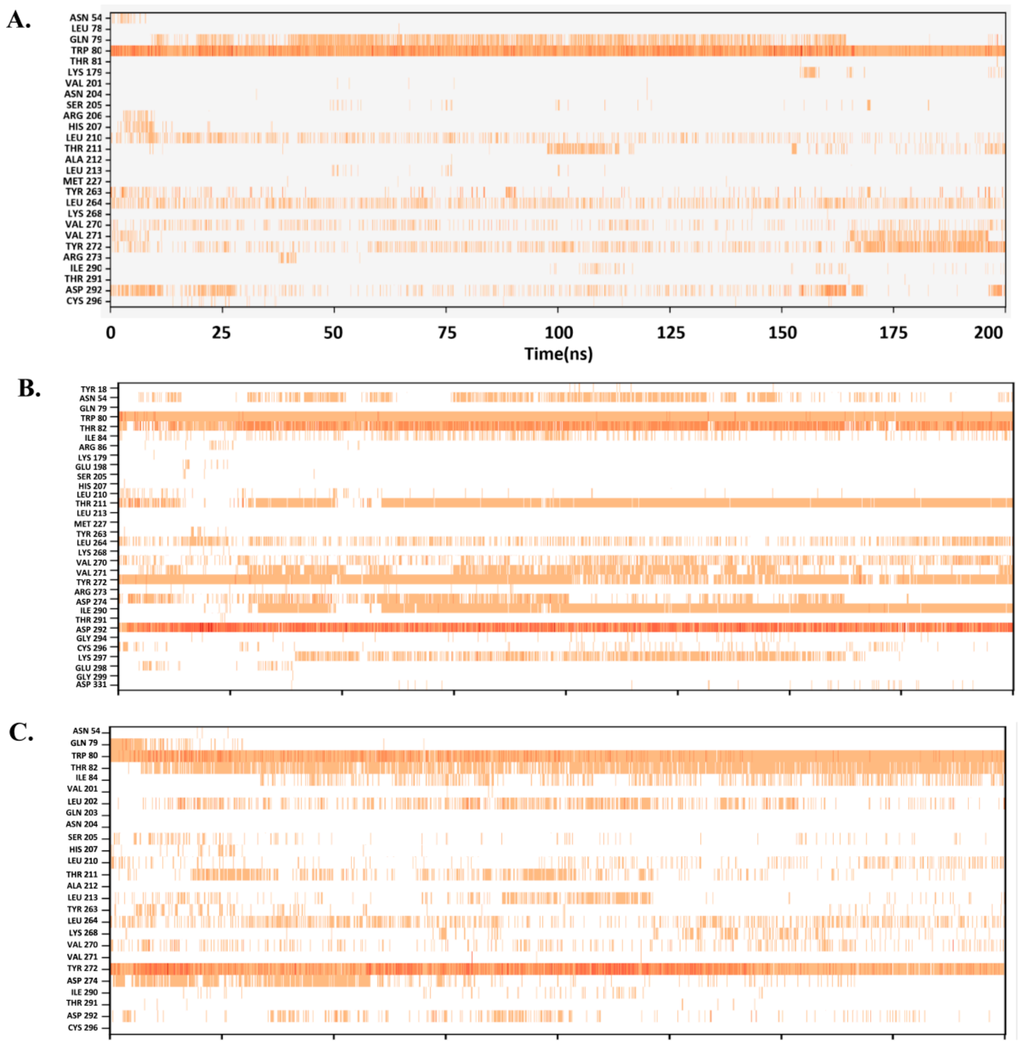

Figure S3: Time-dependent contacts between H-89 and AKT1 of **A)** Run 1, **B)** Run 2, and **C)** Run 3.
